# Technology-Enabled Self-Monitoring of Chronic Obstructive Pulmonary Disease With or Without Asynchronous Remote Monitoring: Protocol for a Randomized Controlled Trial

**DOI:** 10.2196/13920

**Published:** 2019-08-19

**Authors:** Vess Stamenova, Rebecca Yang, Katrina Engel, Kyle Liang, Florence van Lieshout, Elizabeth Lalingo, Angelica Cheung, Adam Erwood, Maria Radina, Allen Greenwald, Payal Agarwal, Aman Sidhu, R Sacha Bhatia, James Shaw, Roshan Shafai, Onil Bhattacharyya

**Affiliations:** 1 Women's College Hospital Institute for Health System Solutions and Virtual Care Women's College Hospital Toronto, ON Canada; 2 Respiratory Therapy Department Markham Stouffville Hospital Markham, ON Canada; 3 Medicine, Care Transitions, Access & Flow, Respiratory Therapy Markham Stouffville Hospital Markham, ON Canada; 4 Support Services & Transformation Markham Stouffville Hospital Markham, ON Canada; 5 Centre For Respiratory Health Markham Stouffville Hospital Markham, ON Canada; 6 Department of Family and Community Medicine University of Toronto Toronto, ON Canada; 7 University Health Network Toronto, ON Canada; 8 Women's College Hospital Research Institute Women's College Hospital Toronto, ON Canada; 9 Hospital to Home and Community Medical Clinic Markham Stouffville Hospital Markham, ON Canada

**Keywords:** chronic obstructive pulmonary disease, remote consultation, remote monitoring, self-monitoring

## Abstract

**Background:**

Chronic obstructive pulmonary disease (COPD) is the third leading cause of mortality worldwide. Reducing the number of COPD exacerbations is an important patient outcome and a major cost-saving approach. Both technology-enabled self-monitoring (SM) and remote monitoring (RM) programs have the potential to reduce exacerbations, but they have not been directly compared with each other. As RM is a more resource-intensive strategy, it is important to understand whether it is more effective than SM.

**Objective:**

The objective of this study is to evaluate the impact of SM and RM on self-management behaviors, COPD disease knowledge, and respiratory status relative to standard care (SC).

**Methods:**

This was a 3-arm open-label randomized controlled trial comparing SM, RM, and SC completed in an outpatient COPD clinic in a community hospital. Patients in the SM and RM groups recorded their vital signs (oxygen, blood pressure, temperature, and weight) and symptoms with the Cloud DX platform every day and were provided with a COPD action plan. Patients in the RM group also received access to a respiratory therapist (RT). The RT monitored their vital signs intermittently and contacted them when their vitals varied outside of predetermined thresholds. The RT also contacted patients once a week irrespective of their vital signs or symptoms. All patients were randomized to 1 of the 3 groups and assessed at baseline and 3 and 6 months after program initiation. The primary outcome was the Partners in Health scale, which measures self-management skills. Secondary outcomes included the St. George's Respiratory Questionnaire, Bristol COPD Knowledge Questionnaire, COPD Assessment Test, and modified-Medical Research Council Breathlessness Scale. Patients were also asked to self-report on health system usage.

**Results:**

A total of 122 patients participated in the study, 40 in the SC, 41 in the SM, and 41 in the RM groups. Out of those patients, 7 in the SC, 5 in the SM, and 6 in the RM groups did not complete the study. There were no significant differences in the rates of study completion among the groups (*P*=.80).

**Conclusions:**

Both SM and RM have shown promise in reducing acute care utilization and exacerbation frequencies. As far as we are aware, no studies to date have directly compared technology-enabled self-management with RM programs in COPD patients. We believe that this study will be an important contribution to the literature.

**Trial Registration:**

ClinicalTrials.gov NCT03741855; https://clinicaltrials.gov/ct2/show/NCT03741855

**International Registered Report Identifier (IRRID):**

DERR1-10.2196/13920

## Introduction

### Background

Chronic obstructive pulmonary disease (COPD) is a heterogeneous condition encompassing disorders such as emphysema and chronic bronchitis, which causes frequent exacerbations [1]. Exacerbations are events occurring in the natural course of the disease characterized by a change in dyspnea, cough, or sputum production. These changes must be beyond normal day-to-day variations, must have acute onset, and may warrant a change in medication. They also cannot be caused by another underlying condition [2].

COPD is the third leading cause of mortality worldwide [3] and accounts for 24% of hospital admissions and 24% of emergency department (ED) visits in Ontario, Canada; COPD is responsible for the highest percentage (18.8%) of 30-day ED readmissions in Ontario [4]. Canada-wide acute COPD exacerbations account for approximately Can $646 million to Can $736 million per year in hospital-based costs [5].

### Self-Management

Reducing the frequency of exacerbations is both an important patient outcome and a major cost-saving approach. Self-management interventions have demonstrated some benefit in reducing the frequency of exacerbations and hospitalizations [6]. Self-management interventions often include some formal patient education, but in some cases, they simply involve sharing an action plan that the patient is expected to follow. An action plan is a list of instructions on what to do when a patient is experiencing an acute exacerbation of a chronic condition [7]. It is often personalized, generated by a health care provider, and meant to promote self-management [7]. A recent Cochrane review concluded that self-management approaches with action plans are associated with improvements in health-related quality of life (QoL) and a lower probability of hospital admission [8]. Studies have also shown that self-management approaches can increase patients’ self-efficacy [9] and improve disease knowledge [10], but results are variable, with some studies reporting no effect [11].

The respiratory symptoms that patients often track as part of their action plan are generally subjective. Patients are instructed to refer to their action plan when their condition changes. Unfortunately, patients often act on their symptoms too late, when their condition has significantly deteriorated. For example, a study that surveyed patients with COPD across 14 countries [12], demonstrated that over a third of patients take a *wait and see* approach at the onset of exacerbations.

### Technology-Enabled Remote Monitoring

With the advent of digital health and remote monitoring (RM) technologies, there is an opportunity to monitor patients regularly, providing greater insight than their subjective experience of symptoms alone. Traditional RM interventions may require patients to record physiological measures (eg, oxygen saturation and blood pressure) or subjective symptoms (eg, dyspnea and activity tolerance) or both on a daily basis [13,14]. In technology-enabled RM programs, the recorded information is stored on a cloud and may be transmitted to a health care provider on a regular basis. A health care provider can either actively monitor the data or refer to it only when needed. In a typical RM program, a clinical professional (eg, nurse and physician) reviews patient data on a regular basis (often once or twice a week) [13]. Health care providers can also be simply *alerted* or *notified* when a significant change is detected by the system or a specific threshold is exceeded [13]. In these instances, a clinical provider must call the patient to inquire about their status and provide guidance in care (potentially avoiding an in-person clinical visit). In some instances, patients are also called by a health professional once or twice a week, irrespective of their readings [15]. A number of studies have reported on the benefits of RM in COPD. For example, a recent Cochrane meta-analysis concluded that RM has shown promise in reducing acute care utilization and the number of exacerbations in COPD patients [16]. Studies of RM have reported lower emergency admission rates [17-19], up to 50% reductions in inpatient admissions [20], and reductions in length of stay [17,21]. RM can also improve patient knowledge of their condition and self-efficacy. For example, Rixon et al [22] showed that patients reported better emotional functioning and mastery 1 year after the implementation of an RM program.

### Technology-Enabled Self-Monitoring

In a technology-enabled self-monitoring (SM) program, similar to an RM program, patients take their recordings daily but are not actively monitored by a clinician; however, a health care provider may have access to the data, if needed. If there is a change in clinical status, the alerts are communicated to the patient and automated instructions on how to deal with exacerbations are provided [23]. This reduces the burden of work for the clinicians and has the potential to provide more timely feedback to the patients. However, it has been suggested that the effectiveness of RM programs may lie in the ability to interact one-on-one with a health care professional [15] and this aspect is completely removed in an SM program. It is, therefore, important to directly compare the 2 programs.

### Study Objectives

Despite increasing evidence of the effectiveness of SM (some of which are technology-enabled) and RM programs, we are not aware of any studies to date that have directly compared the 2 programs. Many so-called *self-management* programs are remotely monitored [11,13,15,24] and as a result, the effectiveness of a technology-enabled self-management program alone, relative to a technology-enabled RM program, is not clear.

Given the lower staff cost and greater ease of implementing a self-management program, the goal of this study was to compare the effectiveness of implementing a technology-enabled SM program with a technology-enabled RM program in a COPD patient population compared with a standard care (SC) group.

We believed that both intervention programs will lead to improvements in self-management skills and respiratory symptoms relative to the SC program. In addition, patients in the RM group may gain more COPD knowledge than those in the SM group.

## Methods

### Study Design

This was an *open-label* randomized controlled trial of 6-month duration comparing 2 technology-enabled interventions relative to SC. Patients were randomized in a 1:1:1 ratio to 1 of 3 groups: an SM group, a self-managing and RM group, from here on called RM, and an SC group. Both SM and RM were technology-enabled. The study recruitment started in April 2018 and was completed in September 2018. Data collection ended in March 2019.

### Participants

#### Eligibility Criteria

To be included in the study, patients needed to be aged 18 years and older and have a clinical diagnosis of COPD that had been established by their respirologist as per clinical guidelines [1]. Exclusion criteria included a diagnosis of other significant lung disease (eg, interstitial lung disease) or dementia, patients without Wi-Fi internet access in their home, inability to read English (required for filling out the questionnaires), participation in other RM programs, or inability to use the technology because of physical or cognitive impairment.

#### Study Setting

The study was conducted at an approximately 300-bed community-based hospital in Ontario. Recruitment was coordinated internally by a clinical study specialist, who was also a respiratory therapist (RT). Patients were recruited from an outpatient COPD clinic (Centre for Respiratory Health), the private practice of respirologists working at the same clinic, and an outpatient COPD rehabilitation program affiliated with the hospital.

### Trial Intervention

#### Technology

The technology used in the study is the *Cloud DX Connected Health Kit* [25]. This specific technology was chosen as it was made by a local Ontario company; it was fully developed and on the market at the time of the study and it allowed for monitoring oxygen saturation.

All patients in the RM and SM groups were provided with the Cloud DX Connected Health Kit as a tool for SM and asynchronous RM. It comprised a custom tablet computer, a Pulsewave wrist cuff monitor, an oximeter, weight scale, and thermometer. The Pulsewave wrist cuff measures blood pressure, heart rate, and breathing rate and uniquely scans for 7 different cardiac anomalies, including missed beats, delayed beats, premature beats, and amplitude anomalies. All of these devices were optimized to work together, prepaired via Bluetooth, and ready to go out of the box and had been approved by the US Food and Drug Administration and Health Canada. Patients in the 2 intervention groups used the kit to record daily physiological and symptom scores by filling out a digital version of the COPD Assessment Test (CAT) [26] and modified Medical Research Council scale [27]. All Cloud DX data were transmitted to a cloud and patients and physicians interacted with it through a Web-based portal.

#### Intervention Procedures

**Figure 1:**
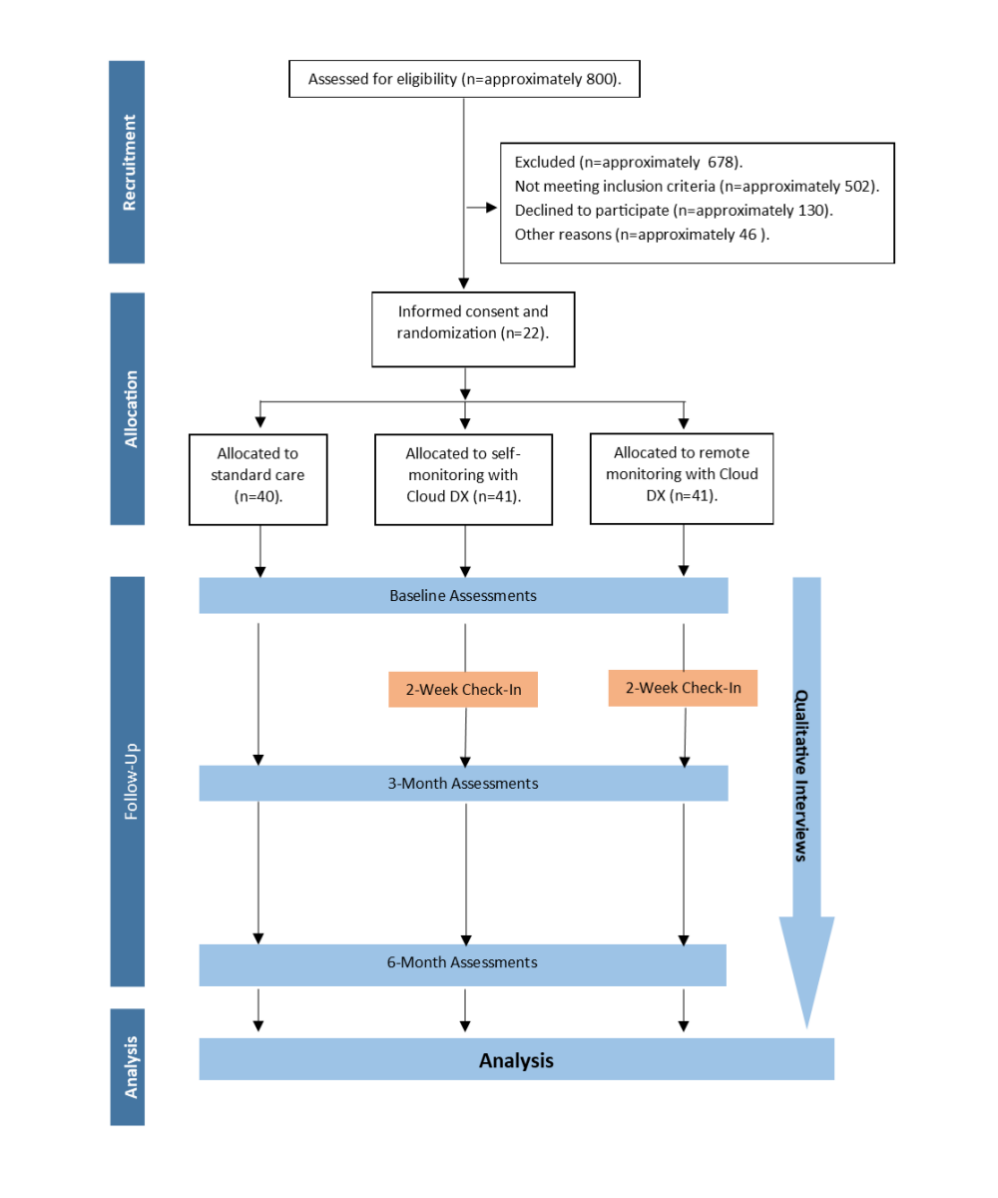
Patient flow through each arm of the study. A total of approximately 800 patients were screened for eligibility to obtain the final sample of 122 participants.

Patients in the SM and RM group received the Cloud DX kit at the start of the trial and continued using it for 6 months. Patients in the SC group were given the opportunity to receive the kit at the end of the trial and use it free of charge and participate in a remote type monitoring program for 6 months (Figure 1).

**Figure 2:**
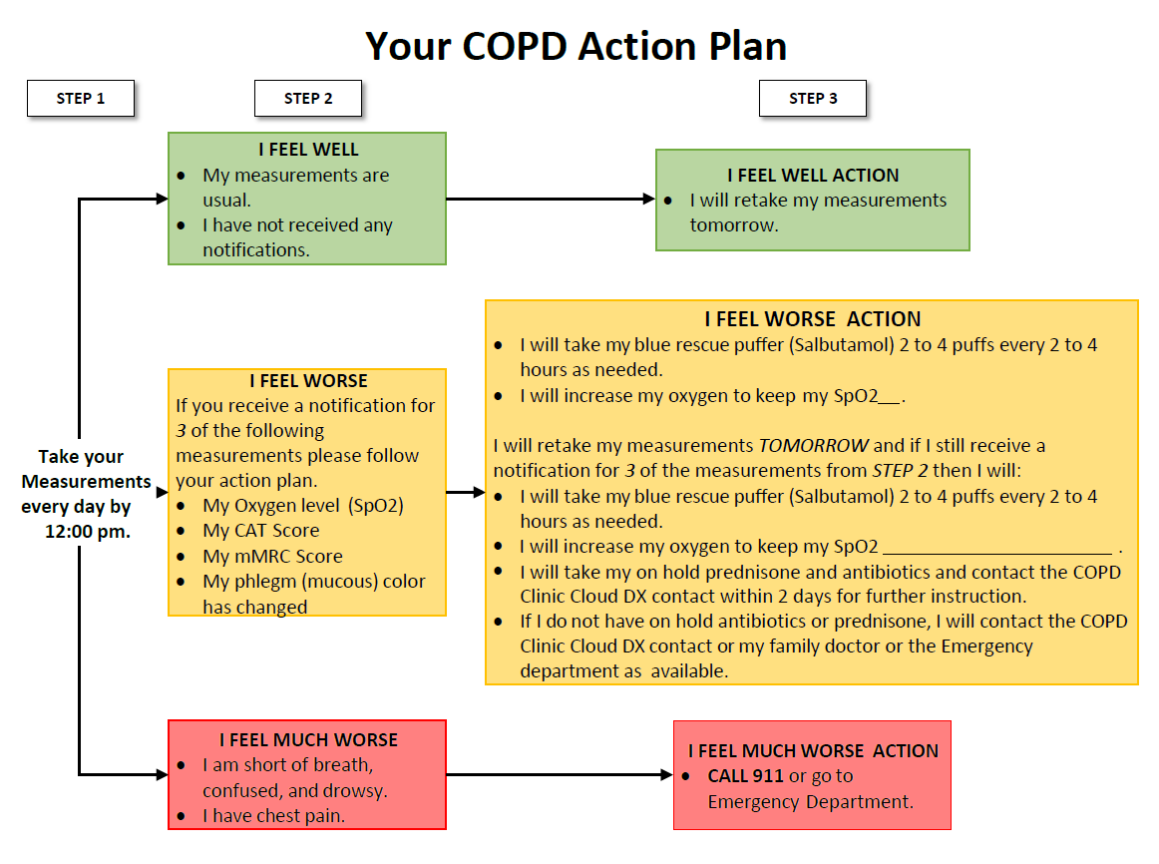
The chronic obstructive pulmonary disease action plan given to participants in the self- and remote monitoring group. The action plan was provided on a piece of paper and patients were asked to refer to it when needed. COPD: chronic obstructive pulmonary disease; CAT: COPD Assessment Test; mMRC: modified Medical Research Council; SpO2: peripheral oxygen saturation.

Patients in the RM and SM group recorded their vitals and symptoms with the Cloud DX platform every day and were provided with a written version of a COPD action plan (Figure 2).

This document comprised a chart that instructed patients on what to do if their readings fell outside the predetermined thresholds. These values were determined on an individual basis by the clinical study specialist in consultation with the patient’s respirologist. The action plan included actions, such as contacting the clinic, filling out a prescription, or going to the ED. Patients in the RM and SM groups also had the option to email or call the clinic with any nonemergency questions they might have had.

For patients assigned to the RM group only, if a patient’s readings fell outside the predetermined thresholds, a notification was sent to both the clinical study specialist and patient through email. Whenever readings exceeded a predetermined threshold, the notifications, along with all other vital signs and symptoms, were reviewed by the clinical study specialist and responded to when clinically indicated. Generally, patients in the RM group received a follow-up call from the clinical study specialist whenever their readings exceeded thresholds twice or more within 2 days. The follow-up calls were completed only Monday to Friday, during regular clinic business hours (8:30 am-4:30 pm). An attempt to complete the follow-up call was done within 24 hours of receiving the notification. If the patient was unavailable, a message was left to call the clinical study specialist back.

The RT also checked on patients in the RM group once a week irrespective of the value of the vitals. The purpose of the call was to check on the patients, prompt action plan use, and act as an educational opportunity to teach the patient about their COPD.

Notifications from the SM group were only sent to the patient. Although the notifications were recorded and were visible to the clinical study specialist, their notifications were not actively monitored or acted upon, unless a patient initiated contact with the clinic.

Aside from these regular (primary) notifications, all patients in the RM and SM group had secondary threshold levels preset by the site investigator for oxygen levels, heart rate, and blood pressure. These were extreme measures that required immediate assistance. Cloud DX staff monitored these levels (as they normally currently do and as is required by Health Canada regulations). In the event that a patient exceeded a secondary threshold, they were contacted by Cloud DX to ensure patient safety and were advised to contact emergency services if they were feeling unwell. Events that required intervention, and were not a result of a technical or reading error, were communicated back to the clinical study specialist. Cloud DX monitored these readings Monday through Friday, 8:30 am to 8:00 pm, and weekends and holidays 9:00 am to 1:00 pm.

All physiological and symptom recordings taken by the patients were transmitted to a secure website where they could be accessed by a predetermined set of the patient’s clinical care providers. The clinical study specialist monitoring the data for the RM group could access the data by looking at the clinician dashboard in this central Web portal, accessible on any personal computer, Mac, or mobile device. Patients in both the SM and RM groups who had not entered data for a week were contacted by Cloud DX staff to inquire whether there was still interest in participation.

Patients in this SC group were not provided with a technology or an action plan, as the action plan was based on vitals and symptoms. This group received the standard care from the respiratory clinic including routine in-person follow-up appointments and access to certified respiratory educator. Some patients may have had an action plan given to them by their respirologists before joining the study, but that action plan was not the same as the one used in the RM and SM groups and was strictly based on subjective symptoms.

The initial visit for RM and SM group patients took 2 hours to allow the obtainment of consent, an introduction of the kit, and baseline survey completion. The initial visit for SC patients took about 1 hour. Patients in the SM and RM groups were also contacted by the clinical study specialist 2 weeks after receiving their kit to reassess the appropriateness of the thresholds. If a revision of the thresholds was required, the clinical study specialist revised it with the respirologist’s approval.

All patients were advised to go to the ED, as they would normally, if they felt the need to at any point in time. Patients were also informed that data were not monitored 24 hours and 7 days a week and to respond to their clinical needs as they would normally do outside of the study.

All patients completed 3 assessments: at baseline, 3 months, and 6 months on a series of questionnaires. Visits 2 and 3 could be done in-person or remotely (on the Web or over the phone). The surveys were available on the Web through a REDCap (Research Electronic Data Capture), Vanderbilt University, electronic data capture tool hosted at Women’s College Hospital [28].

### Outcomes

#### Primary Outcome

The primary outcome of interest was measurement of change in the Partners in Health (PIH) scale [29]. The PIH scale is a validated scale measuring the current status of self-management, with items on knowledge of the condition and skills to monitor and respond to symptoms. This scale was chosen as a primary, as we believed that both interventions could lead to self-management improvement.

#### Secondary Outcomes

The secondary outcomes included measures of general respiratory health, COPD knowledge, and health utilization measures. QoL and respiratory symptoms were assessed with the St. George Respiratory Questionnaire (SGRQ) [30]. The SGRQ is an index designed to measure and quantify health-related status in patients with chronic airflow limitation. It has been shown to correlate well with the established measures of symptom level and disease activity [31]. The first part (*Symptoms*) evaluates symptomatology, including frequency of cough, sputum production, wheeze, breathlessness, and the duration and frequency of attacks of breathlessness or wheeze. The second part has 2 components: *Activity* and *Impacts*. The *Activity* section addresses activities that cause breathlessness or are limited because of breathlessness. The *Impacts* section covers a range of factors including influence on employment, being in control of health, panic, stigmatization, the need for medication, side effects of prescribed therapies, expectations for health, and disturbances of daily life.

The Bristol COPD Knowledge Questionnaire [32] is a measurement of COPD patients’ disease knowledge level. It comprises 13 subscales, each of which assesses a topic of COPD knowledge: (1) epidemiology, (2) etiology, (3) symptom, (4) breathlessness, (5) phlegm, (6) infections, (7) exercise, (8) smoking, (9) vaccination, (10) inhaled bronchodilators, (11) antibiotics, (12) oral steroids, and (13) inhaled steroids. This test has been used in the past to assess patients’ knowledge of COPD [33].

The CAT [26] is a reliable and standardized questionnaire for assessing and monitoring COPD.

Patients were also asked to self-report at baseline, 3 months, and 6 months, the following measures for the past 3 months: the number of COPD-related ED presentations, number of COPD-related admissions to a hospital, the length of stay for all COPD-related admissions (in days), number of exacerbations (episodes in which antibiotics or steroids were prescribed or hospital/clinic visit because of a respiratory issue), number of COPD-related visits to family doctor, number of COPD-related nurse contacts, self-reported use of medication, and self-reported smoking cessation. The number of contacts/calls to the outpatient clinic and deaths were tracked and reported by the clinical study specialist. In addition, hospital admission data and ED use from the local hospital were also obtained.

Finally, vendor-recorded usage data were also documented and sent for analysis at the end of the trial. This included frequency of recordings for oxygen and blood pressure and the number of times thresholds were exceeded.

### Data Analysis Plan

#### Sample Size

One self-management study [34] examined the effects of a telephone self-management program in COPD patients and used both the PIH scale and SGRQ. The effect size of the change over a 6-month period between the control and the intervention group was 0.42 for the PIH and −0.27 for the SGRQ. Assuming an alpha=.05 and correlation between repeated measures of 0.85, a total sample of 82 for a comparison between one of the intervention groups and the SC group at baseline and 6 months (41 patients per group) will produce a power of 0.97 for PIH and 0.71 for the SGRQ. Unfortunately, we did not have the resources to increase the sample size to account for attrition.

#### Recruitment

All eligible patients who were seen at the outpatient COPD clinic within the past year (Centre for Respiratory Health) were contacted for participation and had an equal opportunity to participate (random sampling). Some patients who were seen by the respirologists working outside that clinic were also considered for participation. Some patients were also referred from an affiliated outpatient COPD rehabilitation program that was coordinated by RT working at the COPD clinic. The patients outside the clinic were chosen either by the respirologist/RT as patients that may benefit from the program or self-elected into the study after hearing word of mouth about the study from somebody else.

All eligible patients were contacted by phone, at an appointment, or at the hospital’s exercise rehab program by a clinical staff member (respirologist or RT) who briefly described the technology, provided patients with information about the trial, and requested permission to pass the patient’s information to the clinical study specialist. At this time, the consent form (see Multimedia Appendix 1) was provided to allow patients enough time to consider participation. The clinical study specialist later called the patients to further inquire about their interest in participating and describe the study. The patients who were still interested in the study were scheduled for their baseline evaluation, when the informed consent was obtained, group allocation was revealed, baseline questionnaires were completed, and the Cloud DX kit was provided to them (if in SM or RM group). Patients could split that session into 2, if they felt it was too much for 1 visit.

#### Randomization

Patients were randomized into 1 of the 3 groups using a Web-based random number generator [35]. The generator produced a list of 123 unique numbers from 1 to 123. This list was aligned to a list of group categories. The numbers represented the sequential recruitment of the participant and their corresponding participant ID. The group in which each patient ID belonged was listed on a piece of paper and entered into a sealed envelope with the participant ID on the envelope to allow for allocation concealment from the clinical study specialist who did the recruitment. After obtaining informed consent, the clinical study specialist opened the envelope and determined the group in which the patient was assigned. Once the envelope was opened, both the clinical study specialist and the patient were unblinded to which treatment group the patients were assigned to.

#### Statistical Analysis

All quantitative continuous data will be analyzed by conducting a between-group repeated-measures analysis of variance analyses comparing the scores at baseline, 3 months, and 6 months follow-up assessments of each group. A significant interaction effect between group and time of assessment will indicate that the effect varies per group. Post hoc comparisons will be run to examine changes in outcome between time points. We will also run within-group comparisons, examining differences in performance between each assessment at baseline, 3-month, and 6-month follow-up. Comparison of withdrawal between groups will be conducted and pairwise deletion on missing data will be done (or regression imputation if significant amount is missing).

### Study Significance

The use of technology has the potential to provide clinical marker feedback to the patient and the clinical care provider (RM group), resulting in better disease control and improved self-management skills and QoL. Both programs have the potential to reduce face-to-face visits (outpatient and inpatient admissions). The RM group may be more effective in that regard because of its ability to directly communicate with patients, but it is also more costly in terms of staff time and institutions may incur some additional liability, so it is important to quantify any additional benefit it may provide. The goal was to empower patients and improve their engagement and self-management skills, leading to faster resolution of their health concerns and resulting in fewer complications that may lead to outpatient clinic visits, ED visits, and inpatient admissions. This would increase institutional capacity to offer extended health care services to more patients and improve the overall quality of health care delivered.

### Qualitative Evaluation

A qualitative study about the implementation of the SM and RM programs was embedded during the second half of the study. Interviews were conducted with 8 patients (SM:RM=4:4), 2 caregivers (SM:RM=1:1), 5 health care providers, and 3 hospital administrators/managers who participated in the study or were involved in the implementation of the technology in the clinic. For the patients and health care workers, we aimed to interview both high users and lower users of the technology.

The goal of the qualitative portion was to provide insights in the implementation of the Cloud DX kit in this setting and explore possible improvements for future implementation. Given that technology-enabled programs have not been used in this setting, it was important to understand what worked and what did not and establish what factors could improve the chances for scale-up of the program. The framework tool+team+routine [36] was used for designing the interview guide and analyzing the results. This framework focuses on a *service design* [37] approach to digital health implementation and investigates the changes that need to be made to the tool, team, and routine of everyone involved to create a sustainable delivery of care with successful adoption of the technology. The interviews were audio recorded, transcribed verbatim, and analyzed using thematic analysis [38,39]. The first 2 interviews were coded by multiple researchers and codes were compared to make up the code book for the rest of the interviews to minimize researcher bias.

### Ethics and Dissemination

The study was approved by the Markham Stouffville Hospital and Women’s College Hospital Research Ethics Boards in Ontario, Canada (Protocol version: 1.8, December 7, 2018) (see Multimedia Appendix 2). The study was also retrospectively registered with ClinicalTrials.gov (NCT03741855). Once the results of the study are available, the study will be submitted for publication in a peer-reviewed medical journal and presented at national and international conferences. Significant protocol amendments will be reported to all relevant parties.

### Patient and Public Involvement

During the initial planning stages of the study, we used a co-design approach in the development of the intervention. Patients were given access to the technology for 2 weeks and were subsequently interviewed about their experiences. Health care providers were also interviewed about their current models of care and experience with the technology. The goal of this process was to establish if technology met the needs of its users (patients and health care providers) and determine whether any modifications to the technology and the service it provided were needed. Modifications to both service and technology were done in response to this feedback. Some of this feedback was also used to inform decisions about primary and secondary outcome selection.

Patient advisers were not involved directly in the development of the research question and outcome measures or recruitment. The burden of the intervention was assessed by the research ethics boards who have public member representatives. Any participants interested in receiving information about the results of the study will be provided with a summary once the results are available.

## Results

Data collection is now complete. A total of 122 patients participated in the study, 40 in the SC, 41 in the SM, and 41 in the RM groups. Out of those patients, 7 in the SC, 5 in the SM, and 6 in the RM group did not complete the study because of various reasons (8 withdrew from the trial for various reasons, 6 were noncompliant with their readings, 4 deceased, and 1 dropped because of the technology being difficult to use). There were no significant differences in the rates of study completion among the groups (*P*=.80). We expect the analyses to be completed early in summer and the final version of the report to be submitted for publication before the end of summer.

## Discussion

### Study Contributions

As the digital health field grows in popularity and practice, there are increasing numbers of programs that use digital self-management and RM technologies. Both SM and RM have shown promise in reducing acute care utilization and exacerbation frequencies [6,16]. As far as we are aware, no studies to date have directly compared technology-enabled SM programs with technology-enabled SM plus RM programs. As there is a significant cost associated with having a clinical provider actively monitor the patients’ recordings, it is important to evaluate the impact of a clinician monitoring the data and regularly checking in with patients. This impact will be evaluated as it relates to self-management behaviors, COPD disease knowledge, and respiratory status.

### Strengths

Some of the strengths of this study include the randomized controlled trial design, the ability to not only directly compare the 2 intervention methods but also compare them with SC. Another advantage is that the technology we used is relatively well established and less likely to malfunction in routine use.

### Limitations

The disadvantage of the design was the relatively short assessment period of 6 months. Unfortunately, the funding program that funds the study limited the duration of the intervention. It is possible that certain effects (eg, on health system utilization) will only be detectable over a longer period of time. Finally, the funding available also limited the sample size, and technology interventions sometimes face high dropout rates. Both of these pose a risk to us being able to detect an effect. We have powered the study appropriately for the primary outcome, but it is possible that the secondary outcomes are not adequately powered to rule out an effect. Despite these limitations, we believe that this study will be an important contribution to the literature because it will constitute the first direct comparison of an SM and an RM program.

## Appendix

Multimedia Appendix 1 Approved informed consent form for study participants.

Multimedia Appendix 2 Approved full research protocol for randomized controlled trial (protocol version: 1.8, 7 December 2018).

## Abbreviations

CAT: COPD Assessment Test
COPD: chronic obstructive pulmonary disease
ED: emergency department
PIH: Partners in Health
QoL: quality of life
RM: remote monitoring
RT: respiratory therapist
SC: standard care
SGRQ: St. George Respiratory Questionnaire
SM: self-monitoring
