# Supplementary material for: Technology-Enabled Self-Monitoring of Chronic Obstructive Pulmonary Disease With or Without Asynchronous Remote Monitoring: Protocol for a Randomized Controlled Trial
Source: JMIR Res Protoc. 2019 Aug 19;8(8):e13920. doi: 10.2196/13920 (PMC6718086; doi:10.2196/13920)
Supplement: Multimedia Appendix 1 [file resprot_v8i8e13920_app1.pdf]

INFORMED CONSENT TO PARTICIPATE IN A RESEARCH STUDY

Title of Research Study
Evaluating the Cloud DX platform as a tool for self-management and asynchronous remote-monitoring of chronic obstructive pulmonary disease

Principal Investigators at Women's College Hospital's Institute for Health Systems Solutions and Virtual Care (WIHV), Toronto, ON
Dr. Sacha Bhatia, 416-323-7516, sacha.bhatia@wchospital.ca.
Dr. Onil Bhattacharya, 416-323-6400 ext. 5217, onil.bhattacharyya@wchospital.ca.
Site Investigator at Markham-Stouffville Hospital's Hospital to Home Program, Markham, ON.
Dr. Roshan Shafai, 905-472-7373 ext. 1884, rshafai@msh.on.ca.
Site Research Respiratory Therapist (Main Contact)
Katrina Engel, Clinical Project Specialist, Respiratory Therapy, Markham-Stouffville Hospital, Markham, ON.
905-472-7373, ext. 6958, kengel@msh.on.ca.

Funder
This study is being funded by the Ontario Centres of Excellence Health Technologies Fund.

INFORMED CONSENT
You are being invited to consider participating in a research study. A research study is a way of gathering information on a treatment, procedure or medical device or to answer a question about something that is not well understood.

This form explains the purpose of this research study, provides information about the study device, the test and procedures involved, possible risks and benefits, and the rights of participants.

Please read this form carefully and ask any questions you may have. You may have this form and all information concerning the study explained to you. Please ask the study staff or the investigator to clarify anything you do not understand or would like to know more about. Make sure all your questions are answered to your satisfaction before deciding whether to participate in this research study.

Participating in this study is your choice (voluntary). You have the right to choose not to participate, or to stop participating in this study at any time, and your decision will have no influence on your status at your hospital/institution/employer. If you choose not to participate, you will continue to receive care as usual in the COPD clinic at Markham-Stouffville Hospital (MSH).

INTRODUCTION
You are being invited to consider participating in this study because you have been identified as a patient in Markham-Stouffville Hospital's (MSH) Centre for Respiratory Health, a patient of a private practice clinic of a respirologist affiliated with MSH, or a patient in the Outpatient COPD Exercise Rehabilitation Program with chronic obstructive pulmonary disease (COPD).

Cloud DX is a device, that can help you monitor and manage COPD when you are at home. The device allows you to take your vital readings (including oxygen saturation, blood pressure, temperature, and weight) and complete questionnaires so you have a better understanding of your condition and whether you need to take action. The Cloud DX device and its software application has the potential to improve how you manage your COPD; however, this technology has not been formally evaluated. You are being asked to participate in this research study to assess the value of the Cloud DX device in helping people with COPD to better understand and learn how to cope with COPD.

WHY IS THE STUDY BEING DONE?

The purpose of this study is to examine the impact of the Cloud DX device on helping patients with COPD learn about COPD symptoms and how to respond to the symptom(s) in a helpful way. This in turn has the potential to improve the patient's quality of life. The study also aims to assesses whether monitoring patients with the Cloud DX device improves the efficiency of MSH's Centre for Respiratory Health's COPD clinic.

WHAT WILL HAPPEN DURING THE STUDY?

If you consent to participate, you will receive access to the Cloud DX kit, which includes a tablet, oximeter, blood pressure cuff, weight scale, and thermometer. If you consent, you will also be randomly (by chance) placed in one of three study groups: **remote monitoring**, **self-monitoring**, or **standard care**.

If you are in the **remote monitoring** group, you will complete symptom questionnaires on the tablet and take your readings using the equipment every day for 6 months. Taking these readings should take no more than 15 min/day. The Clinical Project Specialist (who is also a Respiratory Therapist or RT) will work with your respirologist to create a customized action plan that instructs you on what to do in response to the readings. You will receive email notifications if your readings indicate that you are outside of the "normal" range, and you will refer to your action plan to determine the appropriate next-steps. The Clinical Project Specialist will also receive these email notifications during regular business hours (Monday to Friday 8:00am to 4:00pm). The Clinical Project Specialist then will contact you to check in. The Clinical Project Specialist will also give you a weekly call for a general check-in. You will also receive a call from the Clinical Project Specialist 2 weeks after you receive the kit, to review your action plan and to ensure that it has been customized appropriately. Please note that the readings in the remote monitoring group will **NOT** be monitored constantly or regularly. If you feel unwell, please respond to your clinical needs as you would do so normally outside of the study. If you are ever in doubt about whether something is normal or if you are not feeling well, please contact a healthcare professional or go to the emergency department.

If you are in the **self-monitoring** group, you will complete symptom questionnaires on the tablet and take your readings using the equipment every day for 6 months. Taking these readings should take no more than 15 min/day. The Clinical Project Specialist will work with your respirologist to create a customized action plan that instructs you on what to do in response to the readings. You will receive email notifications if your readings indicate that you are outside of the "normal" range, and you will refer to your action plan to determine the appropriate next-steps. The Clinical Project Specialist will **NOT** actively monitor your readings. You will receive a call from the Clinical Project Specialist 2 weeks after you receive the kit to review your action plan and to ensure that it has been customized appropriately. If you feel unwell, please respond to your clinical needs as you would do so normally outside of the study. If you are ever in doubt about whether something is normal or if you are not feeling well, please contact a healthcare professional or go to the emergency department.

If you are in the **standard care** group, you will receive services as you normally would from the COPD clinic at MSH, your respirologist, and/or the Outpatient COPD Exercise Rehabilitation Program. After 6 months, you will receive the Cloud DX kit. You will have 6 months of subscription to the standard Cloud DX services for free, in which you will be able to take your readings and store them. You will also be able to access technical support from Cloud DX. However, the COPD clinic at MSH will **NOT** receive notifications or any other information from the Cloud DX application, you will NOT be provided with a customized action plan, unless your respirologist is willing to provide one. If you feel unwell, please respond to your clinical needs as you would do so normally outside of the study. If you are ever in doubt about whether something is normal or if you are not feeling well, please contact a healthcare professional or go to the emergency department.

Please note that you will need wi-fi access in your home to use the Cloud DX kit. You will be given initial

instructions on how to use the tablet, and will receive support directly from the vendor with regards to any technical concerns or issues that may arise with the Cloud DX kit.

Irrespective of which group you are in, you will also be asked to:

- Complete several questionnaires: at the start of the study, at 3 months, and at 6 months. These can be completed over the phone or on the web. If you wish to communicate by phone you will be able to complete the follow-up questionnaires (at 3 and 6 months) with a member of the research team and they will enter your responses directly in the secure online questionnaire database. Completing the questionnaires should take no more than an hour. You can complete them all at once or stop and continue at a later time (within 1 week).

  Participate in the second (optional) part of the study, which involves an interview to find out from you about your experiences with Cloud DX. The interview will explore whether you believe the application to be useful in your health care and the management of your symptoms. You can choose whether your caregiver is present for your interview. Interviews will take approximately 30-60 minutes each. The research team will do their best to contact you, but they may not be able to follow-up with everyone who expresses interest. You will be asked to sign a separate consent form at the time of the interview

If during the evaluation the device is not working, is lost, stolen or accidently broken, please let the Clinical Project Specialist know as soon as possible. You will not be liable for lost, stolen, or broken tablets. After the study is complete, you will be able to keep the Cloud DX kit. For those in the remote monitoring or self-monitoring groups, you will need to pay a monthly subscription fee of $24.95 after the completion of the study to access of the standard Cloud DX's services. For those in the standard care group, you will receive 6 months of subscription for free following the completion of the study, after which you will need to pay the monthly subscription fee to fully access of the standard Cloud DX's services. If you do not wish to pay the subscription fee, you will still be able to use the oximeter, scale, and thermometer (but NOT the blood pressure cuff), but your readings will not be stored (so you cannot look at your past readings), and you will not have access to Cloud DX technical support. Figure 1: Description of the 3 groups and the assessment timepoints.

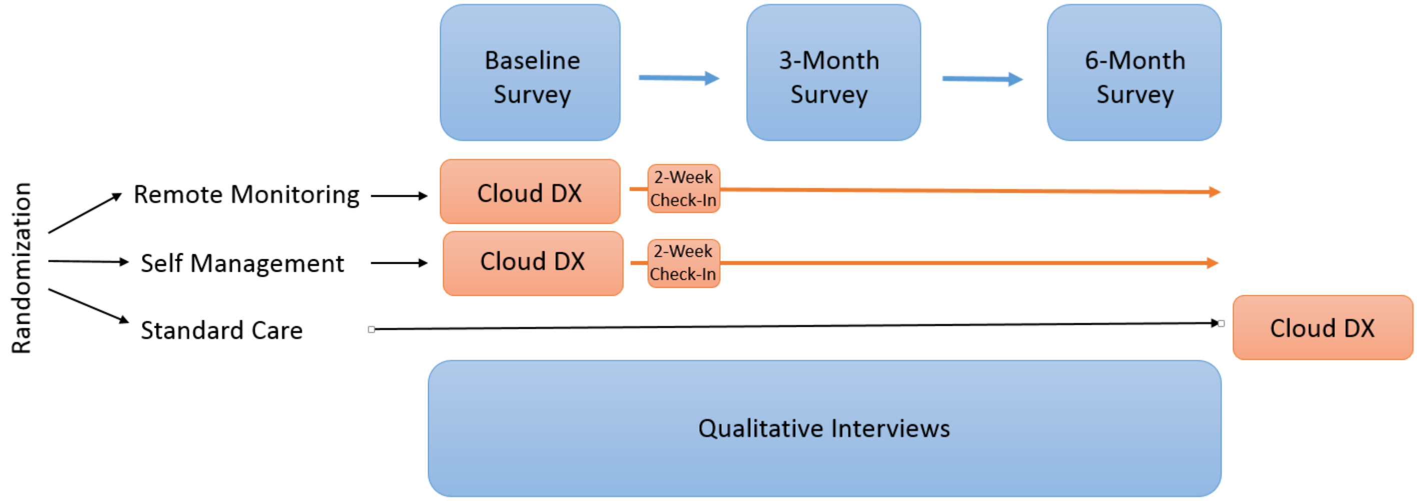

HOW MANY PEOPLE WILL TAKE PART IN THIS STUDY?

It is anticipated there will be a total of 123 participants in this study. Participants will be in the study for 6 months and the study will run at MSH for 1 year.

WHAT ARE THE RESPONSIBILITIES OF STUDY PARTICIPANTS?

If you agree to participate in this study you will be asked to provide the study team access to certain information from your personal and medical records. They may also contact your respirologist, respiratory therapist, or other members of your care team to collect additional information. Your primary care physician will be informed of your participation in this study.

You will be required to complete three sets of questionnaires: at baseline, 3 months, and 6 month. If you would like to complete the surveys online, we will ask for your email address so a direct link can be sent to you at the designated time points. If you are in the remote monitoring or self-monitoring groups, you will be asked to complete the symptom questionnaires on the tablet and take your readings using the equipment every day, and follow your action plan.

WHAT ARE THE RISKS OR HARMS OF PARTICIPATING IN THIS STUDY?

You may feel uncomfortable responding to some of the questions on the surveys, but you can skip questions and stop the survey at any time if that occurs. There is also a risk that you may feel more anxious about your disease when you take your readings and they are below or above the normal threshold. When you first receive the Cloud DX kit, an RT will ensure you know the appropriate steps to take when you receive an abnormal reading.

The research team has established a protocol to ensure there is no harm relating to privacy and confidentiality for participants. Please refer to the *How will my information be kept confidential?* section for more information.

You will be told about any new information that might reasonably affect your willingness to continue to participate in this study as soon as the information becomes available to the study staff. However, please note that participation in this study may involve unforeseeable risks.

If you have questions or concerns about the study, or if you experience an injury as a result of the study, please contact the Clinical Project Specialist directly at 905-472-7373, ext. 6958

If you have an urgent medical issue related to your health care, you should go to the nearest emergency department for assessment.

WHAT ARE THE COSTS OF PARTICIPATING IN THIS STUDY?

Participation in this study will not involve any additional costs to you. If you are required to travel to MSH for the purpose of the study, you will be reimbursed for the parking costs.

WHAT ARE THE BENEFITS OF PARTICIPATING IN THIS STUDY?

You may or may not benefit from this study. However, you may benefit from accessing a virtual care tool that aims to improve the monitoring and management of your COPD. You are also able to keep the Cloud DX kit after the completion of the study.

ARE STUDY PARTICIPANTS PAID TO PARTICIPATE IN THIS STUDY?

You will not be paid money to participate in this study. However, you are able to keep the Cloud DX kit after the completion of the study and take readings with all devices, except the blood pressure cuff.Keep in mind that for full device support and to use the blood pressure cuff you will need to pay a monthly subscription fee of $24.95.

HOW WILL MY INFORMATION BE KEPT CONFIDENTIAL?

- To ensure privacy and confidentiality remain intact, the study team will ensure that information is "de-identified": all participants are assigned a code, which will be used in place of their names to maintain confidentiality. The information cannot be linked back to you.
- All identifying information is destroyed following data analysis. That means no information will be released or printed that would disclose personal identity.
- No subject identifiers of participants is included in the conduct, data storage, analysis, and presentation

of findings (data will be presented in aggregate form only).
- Consent forms of study participants is stored separately from the data files (transcripts and analytical worksheets).

You have the right to have any health information about you that is collected, used or disclosed for this study to be handled in a confidential manner. You also have the right to access, review and request changes to your personal health information

Access to your personal health information will take place under the supervision of the Principal Investigator. However, only Cloud DX and the Clinical Project Specialist will have access to information that identifies you. The Principal Investigator and study staff will only have access to "study data". "Study data" is information about you that is collected for the study, but that does not directly identify you. The study data will be assigned a code and will be added to the study data from all other study participants and sent securely to Women's College Hospital after study completion for the research purposes explained in this form.
The investigator(s), study staff and the other people listed above will keep the information they see or receive about you confidential, and, to the extent permitted by applicable laws/regulations, will not make it publicly available. Even though the risk of identifying you from the study data is very small, it can never be completely eliminated. The Principal Investigator will keep any personal health information about you in a secure and confidential location for 10 years after publication and then destroy it according to Women's College Hospital and Markham-Stouffville Hospital policy. When the results of this study are published, your identity will not be disclosed.

Cloud DX will be provided access to your name and contact information (email address, telephone number, and home address), , along with data around things like how often you used the Cloud DX platform so that they may improve their product. The data you enter on the tablet will also be stored indefinitely on secure Cloud DX servers that are compliant with health Canada regulations and loaded every time you log on. Nothing is stored on the tablet.

Description of this clinical trial will be available on http://www.ClinicalTrials.gov, as required by US Law. This website will not include information that can identify you. At most the Web site will include a summary of the results. You can search for this website at any time.
The following people may look at your personal health information to check that the information collected for the study is correct and to make sure the study followed the required laws and guidelines:

- Members of the research study team at the Women's College Hospital, Markham-Stouffville Hospital, or their representatives in your area;
- Representatives of the Women's College Hospital, Women's College Hospital Research Institute, or the Women's College Hospital Research Ethics Board, because they oversee the ethical conduct of research studies at Women's College Hospital

DO THE INVESTIGATORS HAVE ANY CONFLICTS OF INTEREST?
There are no conflicts of interest to declare related to this study.

WHAT ARE THE RIGHTS OF PARTICIPANTS IN A RESEARCH STUDY?
You have the right to receive all information that could help you make a decision about participating in this study. You also have the right to ask questions about this study and your rights as a research participant, and to have them answered to your satisfaction, before you make any decision. You also have the right to ask questions and to receive answers throughout this study. You have the right to withdraw from the study at any time. The data that has already been accumulated will remain as part of the research study. However, if you would like your de-identified study data removed from the study, please let us know.

You have the right to be informed of the results of this study once the entire study is complete. If you would like to be informed of the results of this study, please indicate in the "Documentation of Informed Consent" section at the end of this form that you would like to be contacted.

In no way does signing this form waive your legal rights nor release the study doctor(s), sponsors or involved institutions from their legal and professional responsibilities.

CAN THE INVESTIGATORS WITHDRAW ME FROM THE RESEARCH STUDY?

You may be withdrawn from the study in the event that you are no longer living in the community, if you stop receiving care from the Markham-Stouffville Hospital's (MSH) Centre for Respiratory Health or if you develop a condition that is fits the study exclusion criteria. In the case of a withdrawal from the study, you will keep the Cloud DX technology.

If you have any questions about this study it is best to contact the Clinical Project Specialist, Katrina Engel, Respiratory Therapy, Markham-Stouffville Hospital, Markham, ON. 905-472-7373, ext. 6958, kengel@msh.on.ca.

You may also contact one of the two people in charge of this study, Dr. Sacha Bhatia, at the Women's College Hospital, at 416-323-7516; or Dr. Onil Bhattacharyya, at Women's College Hospital, at 416-323-6400 ext. 5217.

You may also contact the Chair of the Women's College Hospital REB, Dr. Nancy Walton, at 416-351-3732 ext. 2325, or the Chair of Markham-Stouffville Hospital REB, Dr. Henry Solow, at 905-472-7072.

DOCUMENTATION OF INFORMED CONSENT

A copy of this informed consent form will be sent to you by email after you have given your verbal approval or given to you at your next visit by the study staff.

Full Study Title: Evaluating the Cloud DX platform as a tool for self-management and asynchronous remote-monitoring of chronic obstructive pulmonary disease

Name of Participant: ____

Participant/Substitute decision-maker
By signing this form, I confirm that:

- This research study has been fully explained to me and all of my questions have been answered to my satisfaction
- I understand the requirements of participating in this research study
- I have been informed of the risks and benefits, if any, of participating in this research study
- I have been informed of any alternatives to participating in this research study
- I have been informed of the rights of research participants
- I have read each page of this form
- I authorize access to my personal health information, medical record, and research study data as explained in this form
- (If applicable) I have agreed, or agree to allow the person I am responsible for, to participate in this research study
- I understand that my primary care provider may be informed of my participation in this research study

____________________________	________________________	______________________
Name of Person Assisting (Print)	Signature	Date

The following components are optional and not required in order to participate in the main study.

☐ I **agree** to be contacted to complete the second part of this study which involves an interview process to find out from me, about the Cloud DX application as described in this consent form. If you agree to be contacted for the second part of the study, you will need to engage in a separate consent process. You may change your mind about this at any time.

Please indicate your preferred method of contact:

☐ Email ☐ Phone

Email or Telephone: ___________________________
(If phone) You may leave a voice message: ◻ YES or ◻ NO
Would you like to be informed of the study results? ◻ YES or ◻ NO
Preferred method of contact for study results:
◻ Email ◻ Phone

ASSISTANCE DECLARATION
Was the participant assisted during the consent process? ◻ YES or ◻ NO
□ The consent form was read to the participant, and the person signing below attests that the study was accurately explained to, and apparently understood by, the participant.

_____________________________ ________________________ ______________________
Name of Person Assisting (Print) Signature Date

Person obtaining consent
By signing this form, I confirm that:
• This study and its purpose has been explained to the participant named above
• All questions asked by the participant have been answered
• I will give a copy of this signed and dated document to the participant

_________________________ ________________________ ______________________
Name of person obtaining consent (Print) Signature Date

If applicable:
□ Consent obtained by telephone ☐ Hard-copy mailed/emailed to participant,
Date: _______________________ Initial: ________
