# Supplementary material for: Technology-Enabled Self-Monitoring of Chronic Obstructive Pulmonary Disease With or Without Asynchronous Remote Monitoring: Protocol for a Randomized Controlled Trial
Source: JMIR Res Protoc. 2019 Aug 19;8(8):e13920. doi: 10.2196/13920 (PMC6718086; doi:10.2196/13920)
Supplement: Multimedia Appendix 2 [file resprot_v8i8e13920_app2.pdf]

**Title:** Evaluating the Cloud DX platform as a tool for self-management and asynchronous remote-monitoring of Chronic Obstructive Pulmonary Disease

## 1. Background and rationale

Chronic obstructive pulmonary disease (COPD) is a pervasive disease that is estimated to effect 2.6 million Canadians (17%) aged 35 to 79 have COPD (Government of Canada, 2014) costing the health care system in Canada 1.5 billion annually (Mittmann et al., 2008). Ontarians living with COPD are frequent users of the health care system and account for 24% of hospital admissions and 24% of emergency department (ED) visits (Gershon, Guan, Victor, Goldstein, & To, 2013) and COPD is responsible for the highest percentage (18.8%) of 30-day readmissions to ED in the province (Government of Canada, 2017).

It has been demonstrated that sources of care beyond the hospital (e.g. home care or outpatient clinics) that are aligned with a comprehensive discharge plan are effective ways of reducing hospital readmissions for COPD (Ko et al., 2014). As a result, care for COPD patients has been moved to the community and local health care clinics, such as Markham Stouffville Hospital (MSH), who are tasked with follow up care for COPD patients. However, access to these services remains a challenge due to funding restrictions. For example as per our communication with the hospital, the outpatient clinic can see no more than 600 patients per year, which places pressure on patients to self-manage their condition with limited supports. Local community centres are often tasked with providing support to self-manage COPD at home, but limited resources makes it challenging and there is a need to optimize self-management.

*Self-management* is a person's ability to manage the clinical and psychosocial effects of their chronic condition and their ability to make lifestyle adaptations inherent in living with a chronic condition (McBain, Shipley, & Newman, 2015). A recent Cochrane review of self-management interventions in COPD (face-to-face, not technology based and not studies that involved physiological recordings) concluded that they are associated with improved quality of life and a reduction in hospital admissions (Zwerink et al., 2014). A foundational component of self-management is "self-monitoring", defined as "the patient undertaking one or more of the following activities (i) self-measurement of vital signs, symptoms, behaviour or psychological well-being; (ii) self-interpretation of this data; or (iii) self-adjustment of medication, treatment, lifestyle or help-seeking behaviour as a result of self-awareness and/or self-interpretation (McBain et al., 2015). COPD patients often have difficulty detecting early deterioration in their symptoms. Self-monitoring programs may provide the means of monitoring patients' in the community allowing for detection of deteriorations quicker and for an intervention before significant deterioration occurs (Rojahn et al., 2016).

*Technology-enabled* self-monitoring still requires patients to take self-measurements, but also has the potential to remove the self-interpretation and self-adjustment components of self-monitoring from the responsibility of the patient. This can be achieved by the technology (e.g. a tablet software program) registering the readings of the patients, interpreting the data and providing automatic feedback to patients about the type of adjustments they need to make. This feedback has the potential to allow patients to learn about their condition and to recognize their symptoms early on. Currently, most technology-enabled self-monitoring programs also transmit the data to a healthcare professional who monitors the data remotely (*remote-monitoring*) and contacts the patient when needed(Cruz, Brooks, & Marques, 2014).

The benefits of remote monitoring are quite well established. A recent Cochrane meta-analysis concluded that remote monitoring has shown promise in reducing acute care utilization and

reducing the number of exacerbations in COPD patients (McLean et al., 2012). Studies of remote monitoring have reported lower emergency admission rates (De San Miguel, Smith, & Lewin, 2013; Pedone, Chiurco, Scarlata, & Incalzi, 2013; Steventon et al., 2012), up to 50% reductions in inpatient admissions (Brown Connolly, 2014) and reductions in length of stay (Clarke, Fursse, Connolly, Sharma, & Jones, 2017; De San Miguel et al., 2013). Remote monitoring can also lead to improved patient's knowledge and self-efficacy. For example, Rixon et al. (2015) showed that patients reported better emotional functioning and mastery 1 year after the implementation of a remote monitoring program.

Some have argued, however, that the benefits of self-monitoring programs lie in the remote-monitoring component (the direct contact with a healthcare provider) (McBain et al., 2015) The authors acknowledge, however, that this has not systematically been studied, especially in the COPD patient population. Furthermore, this has not been studied in a *technology-enabled* self-monitoring program that provides feedback to patients.

Furthermore, technology-enabled self-monitoring programs with patient feedback have a great cost-saving potential. As such, remote monitoring is expected to reduce healthcare utilization and cost (Haesum et al., 2012). However, preliminary studies on the cost-effectiveness of these programs have provided mixed results. Some studies have suggested that it can lead to reduction in costs (De San Miguel et al., 2013), while others have reported that the cost-effectiveness of the programs is not clear (Clarke et al., 2017). One significant cost associated with remote monitoring programs is the labor cost associated with paying a clinical health provider to monitor patients' recordings. Self-monitoring does not involve remote monitoring and as such its cost per patient is lower than remote-monitoring. If technology enabled self-monitoring with patient feedback can be demonstrated to be as effective as remote monitoring, then the savings for administering such a program can be substantially reduced.

In summary, technology-enabled self-monitoring programs have the potential to educate patients about their condition, teach them how to self-manage and have the potential to significantly reduce costs.

## 2. Research Objectives

Therefore, the goal of the current project is to investigate the effectiveness of implementing a technology-enabled self-monitoring program (SM group) or a technology enabled self- and remote-monitoring program (RM group) in a COPD patient population compared to standard of care. Effectiveness will be measures by outcomes examining patients' self-management skills, quality of life, COPD knowledge and respiratory symptoms. The standard of care group (SC group) currently does not use any technology to monitor patients at their home. Therefore the following 3 conditions will exist in the study:

1. SM group: Patients in this group will be recording their vitals and symptoms with the Cloud DX platform everyday and will be provided with an action-plan that instructs them on what to do in response to their readings.
2. RM group: Patients in this group will be recording their vitals and symptoms with the Cloud DX platform everyday and will be provided with an action-plan that instructs them on what to do in response to their readings. In addition, a respiratory therapist (RT) will be monitoring asynchronously their vitals and contacting them when their vitals exceed pre-determined thresholds. The RT will also check on patients once a week irrespective of the value of the vitals.

3. SC group: patients will not be provided with a technology or an action plan (as the action plan is based on vitals and symptoms).

## 3. Trial design

We will use a mixed methods design for this study, containing both a quantitative and a qualitative component. The quantitative component is presented first and the qualitative component follows. An open-label randomized controlled trial will be used for this study. Patients will be randomized to one of 3 groups: a RM, a SM intervention group and a SC group (41 patients per group).

## 4. Methods

### a. Study Setting

The study will be conducted at Markham Stouffville Hospital (MSH) with most patients being recruited from the Outpatient COPD Clinic (Respiratory Health-COPD Clinic).Recruitment will be coordinated internally by the clinical project specialist, who is also a respiratory therapist. Some patients will also be identified through inpatient admission records and the Health for All Family Health Team (FHT) by their family doctors who have clients that are living in the community with diagnosed COPD Additional patients will be recruited by respirologists. These respirologists practice at the Outpatient COPD clinic, but also have separate private practices in Markham. Finally, patients will be recruited from the Outpatient COPD Exercise Rehabilitation Program that is run at the Cornell Community Centre in collaboration with MSH. These patients will be approached by an MSH respiratory therapist who coordinates the rehabilitation program. We have already established a research collaboration with the hospital during the design phase of this project that informed our methods and recruitment practices. Access to potential patients will be determined locally, in-house at MSH by the clinical project specialist, who is also a respiratory therapist. All data collection will be done internally at MSH. All initial contact will be done internally to the site from where the patient is originating (e.g. Outpatient COPD Clinic, COPD rehabilitation program, Family Health Team (FHT), or a respirologists' private practice). For sites that are outside MSH, patients will be provided with the brochure for the study and will be asked for permission to be contacted by the clinical project specialist.

### b. Eligibility criteria

To be included in the study patients need to be at least 18 years of age and have a clinical diagnosis of COPD that has been established by their respirologist as per clinical guidelines (Dewar & Whit R. Curry, 2006). Exclusion criteria include a diagnosis of other significant lung disease (e.g. interstitial lung disease) or dementia, patients without wifi at their home, patients who do not speak English (required for filling out the questionnaires) or patients unable to use the technology due to physical or cognitive impairment.

### c. Recruitment

Recruitment will be on-going until 123 patients have been recruited for the intervention phase (see sample size justification in Section 6.b). Patients will be approached by the clinical staff (respirologist or respiratory therapist-RT) who will introduce the technology to them and provide them with brief information about the trial with a brochure (Appendix 19) and request permission to pass the patient's information to the clinical project specialist at MSH. At this time the consent

form will also be given to give the patients enough time to consider participation. The clinical project specialist will later call the participants to further inquire about their interest in participating and to provide further details about the project. At this time they will also confirm the patient has a copy of the consent form and if they don't have the clinical project specialist will email it to the patient. The patients who are still interested in the project will be scheduled for their baseline evaluation, at which time informed consent will be signed (Appendix 1) and the Cloud DX kit will be provided to them. Patients can split that session into two, if they feel it is too much for one visit. There will also be a recruitment video that will be uploaded on YouTube. The clinical project specialist will email the link with the brochure to interested patients. The clinical project specialist will also be showing the video on a tablet to patients in the Outpatient COPD Exercise Rehabilitation Program. The Inpatient RT will show the video on a tablet to patients with COPD prior to their discharge from hospitalization.

d. Intervention

Patients will be randomized to either SM, RM group or SC group (41 patients per group). Patients in the SM and RM group will receive the Cloud DX kit at the start of the trial and will continue using it for 6 months. Patients in the SC group will be given the opportunity to receive the kit at the end of the trial and use it for free for 6 months (see Figure 1). All patients will complete 3 assessments: at baseline, 3 months and 6 months on a series of questionnaires (see Outcome section for a list and description). They will also complete a demographic questionnaire at the initial visit (Appendix 13). The clinical project specialist at MSH will be responsible for collecting all data at each visit. The initial visit is expected to take 2 hours, allowing for obtaining consent, an introduction of the kit, and survey completion. Subsequent visits are expected to take about 1 hour to complete, as only surveys will be filled out. Visit 2 and 3 will be done in-person or remotely as surveys will be completed through REDCap and patients will have the option to complete them online from their home or over the phone. The clinical project specialist will make contact with the patient via telephone to remind them of the survey deadline. If patients prefer to come in person, they will be provided with that option. If the patient prefers to conduct the survey over the phone, they will be transferred over to the research team at WCH with a script (Appendix 20). Once the patient verbally agrees, the clinical project specialist at MSH will call one of the Research Assistants at WCH to provide the patient name, telephone number, and email address (if applicable). If the Research Assistant does not pick up, a voice message will be left on the Research Assistant's password protected mailbox. The Research Assistant then documents patient information in a password protected Excel document in the WCH internal drive. The Research Assistant will then contact the patient to conduct the survey as well as email the patient for any follow-up requests from the phone call, ie. to resend the REDCap survey link.

Patients in the SM and RM group will also be contacted by the clinical project specialist 2 weeks after receiving their kit to review whether their thresholds have been set properly. If a revision of the thresholds is needed this will be done with the respirologist's approval.

In addition, the clinical project specialist at Markham-Stouffville Hospital will conduct a chart review to collect patient characteristics listed in the Appendix 21 - Patient Characteristics Collected from Chart Review.

Figure 1. Patient flow through the study.

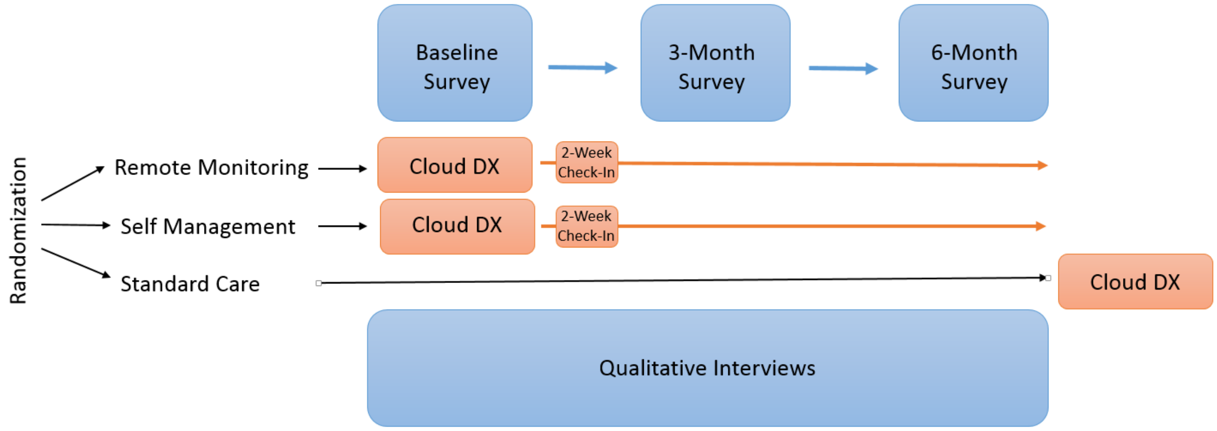


## The Cloud DX Kit

The technology we will use in the study is the Cloud DX Connected Health Kit. All patients will be provided with the Cloud DX Connected Health Kit as a tool for self-monitoring and asynchronous remote monitoring. It consists of a custom Tablet computer, Pulsewave® wrist cuff monitor (Health Canada license, Appendix 2), Oximeter (Health Canada license, Appendix 3), Scale and Thermometer (Health Canada license, Appendix 4). Cloud DX is also a licensed distributor of medical devices (MDEL: 6984, Appendix 5). Patients are also provided with a comprehensive user manual (Appendix 6). All of these devices are optimized to work together, pre-paired and ready to go out of the box. All patients will use the kit to record daily their physiological and symptom readings. All patients will also answer general questions about their symptoms, by filling out a digital version of the COPD Assessment Test (CAT, Appendix 7) (Jones et al., 2009) and MRC scale (Stenton, 2008) (Appendix 8). The Pulsewave wrist cuff measures blood pressure, heart rate, breathing rate and detects cardiac anomalies with comfort and ease of use.

All patients in the RM and SM group will also be provided with an action plan document that will be given on a piece of paper. This document consists of a chart that instructs patients on what to do if their readings fall outside their respirologist pre-specified values. The action plan includes actions such as calling the clinic, filling out a prescription or going to the emergency department (see Appendix 17).

For patients assigned to the RM group, if a patient's readings fall outside the pre-determined thresholds, a notification will be sent to the clinical project specialist at MSH and to the patient through email notifying them that the reading exceeded the threshold. Patients in the RM group will also receive a follow-up call from the clinical project specialist whenever their readings exceed thresholds. The follow-up calls will be done only Monday to Friday 9-5. An attempt to complete the follow-up call will be done within 24 hours of receiving the notification and a message will be left to call back the clinical project specialist if patients are not available when that call is made.  The purpose of the call is to check on the patients, to encourage them to follow their action plan and to use the calla s an educational opportunity to teach patients about

COPD as their questions arise. Patients in the RM group will also have the option to send a message to the clinic with any non-emergency questions they may have.

Notifications in the SM group will be recorded and send to the clinic, but their notifications will not be actively monitored or acted upon.

Aside from these regular (primary) notifications, all patients (both SM and RM group) will have a secondary threshold levels preset by their respirologist for oxygen levels, heart rate and blood pressure. These will be extreme measures that require immediate assistance. Cloud DX staff will monitor these levels (as they normally currently do and are required by Health Canada regulations). In the event that a patient exceeds a secondary threshold, they will be contacted by Cloud DX, to ensure that everything is ok and advised to contact 911. All of these events will be communicated back to the clinical project specialist. Cloud DX monitors these readings Mon-Friday 830am-800pm and weekends and holidays 9am-1 pm.

All patients will be advised to go to the emergency department as they would normally if they feel they have the need to. Patients will also be informed that data is not monitored 24hrs/7 days a week and if they need care immediately to respond to their clinical needs as they would normally do outside of the study.

All physiological and symptom recordings taken by the patients will be transmitted to a secure Web site where they can be accessed by a pre-determined set of the patient's clinical care providers. The clinical project specialist at MSH will monitor each patient's recordings once a week and receive notifications when a patient's readings exceed the specific thresholds. The employee will screen any notifications or messages received once a day Mon-Fri. Each patient will have a personalized threshold level for their vitals that will be based on a threshold predetermined by the respirologist. The clinical project specialist monitoring the data for the RM group can access the data by looking at the Clinician Dashboard in this central web portal, accessible on any PC, Mac or mobile device. By monitoring blood pressure, pulse oxygen, temperature and body weight and using smart surveys, clinicians at MSH will have a better idea of the level of control each patient has over their COPD; which patients need the most help adjusting to their COPD management plan at home; and which COPD patients need to be seen in a community setting, maximizing scarce resources. Patients in the RM and SM groups will be instructed to refer to their action plan with each reading and follow the flow chart based on their symptoms and readings. The data of the patients in the RM group will also be screened at the clinic once a week, even if no notifications have been sent. Patients will be informed that their data will not be screened constantly and if they feel unwell, they should contact their clinical provider as they would do normally or go to the emergency room if they feel they need to see a doctor immediately or if the clinic is closed. Participants who have not entered data for a week will also be contacted by the clinical project specialist to inquire whether there is still interest in participation.

e. Outcomes

**i. Primary Outcome**

- *The Partners in Health (PIH) scale* (Petkov, Harvey, & Battersby, 2010) (Appendix 9).A validated scale measuring the current status of self-management, with items on knowledge of the condition and skills to monitor and respond to symptoms.

**ii. Secondary Outcomes**

- Quality of Life and Respiratory symptoms will be assessed with the *St. George's Respiratory Questionnaire (SGRQ)* (Jones, Quirk, & Baveystock, 1991) (Appendix 10).

The St. George's Respiratory Questionnaire (SGRQ) is an index designed to measure and quantify health-related health status in patients with chronic airflow limitation. It has been shown to correlate well with established measures of symptom level, disease activity and disability. The first part ("Symptoms") evaluates symptomatology, including frequency of cough, sputum production, wheeze, breathlessness and the duration and frequency of attacks of breathlessness or wheeze. The second part has two components: "Activity" and "Impacts". The "Activity" section addresses activities that cause breathlessness or are limited because of breathlessness. The "Impacts" section covers a range of factors including influence on employment, being in control of health, panic, stigmatization, the need for medication, side effects of prescribed therapies, expectations for health and disturbances of daily life.

- *Bristol COPD Knowledge Questionnaire* (White, Walker, Roberts, Kalisky, & White, 2006) (Appendix 18) is a measurement of COPD patients' disease knowledge level. It consists of 13 subscales, each of which assesses a topic of COPD knowledge: (1) epidemiology, (2) aetiology, (3) symptom, (4) breathlessness, (5) phlegm, (6) infections, (7) exercise, (8) smoking, (9) vaccination, (10) inhaled bronchodilators, (11) antibiotics, (12) oral steroids, and (13) inhaled steroids. This test has been used in the past to assess patients' knowledge of COPD(Choi, Chung, & Han, 2014; Zhang et al., 2014).
- The *COPD Assessment Test* (Jones et al., 2009) (Appendix 7)
- *MRC Breathlessness Scale* (Stenton, 2008) (Appendix 8)
- Patients will also be asked to self-report at baseline, 3m and 6m the following measures for the past 3 months: the number of ED presentations, number of admissions to a hospital, the length of stay for all admissions (in days), number of exacerbations (episodes in which antibiotics or steroids were prescribed or hospital/clinic visit due to a respiratory issue), number of visits to family doctor, number of nurse contacts, self-reported use of medication, self-reported smoking cessation (Appendix 13)
- Number of contacts/calls to the outpatient clinic and deaths will also be kept track of by the clinical project specialist at MSH

## Data collection, management, and statistical analysis

All quantitative continuous data will be analysed by conducting a between-group repeated-measures ANOVA analyses comparing the scores at baseline, 3m and 6m follow-up assessments of each group. We will also run within-group comparisons, examining differences in performance between each assessment at baseline, 3m and 6m follow-up. All analysis will be run by a statistician at WIHV.

f. Sample Size

One self-management study (Walters et al., 2013) examined the effects of a telephone self-management program in COPD patients and used both the PIH scale and SGRQ. The effect size of the change over a 6m between the control and the intervention group was 0.42 for the PIH and -0.27 for the SQHQ. Assuming an $\alpha=0.05$, and correlation between repeated measures of 0.85, a total sample of 82 for a comparison between one of the intervention groups and the standard care group at baseline and 6m (41 patients per group) will produce power of 0.97 for PHI and 0.71 for the SGHQ. It is not clear whether we'd see any changes at 3m, but we are confident that we'd detect some effects at 6m.

g. Data Collection

All assessment data will be collected by the clinical project specialist at MSH and entered into a de-identified database that will be then sent to the research coordinator at Women's College Hospital. The clinical project specialist will also assign a Cloud DX ID that will be distinct from the REDCap ID. REDCap IDs will be consecutive, while Cloud DX IDs will be random, so that they cannot be easily linked to each other. All data will be entered into REDCap by the clinical project specialist.

Data collected through the chart review will be collected and tracked using the Health Care Utilization Chart Review Tool (Appendix 22 - COPD Cloud DX Health Care Utilization Chart Review Tool).

h. Data Management

All data will be stored on password protected computers accessible only by members of the research team in locked rooms at Women's College Hospital. All data will be gathered from original sources, including the Cloud DX data management system, and directly from patient surveys or interviews and transferred to Women's College Hospital for storage through REDCap. The participants will be identified only through a participant ID number. The information will be encrypted and will be accessed by the study team through a secure VPN. A data sharing agreement which will detail how data will be transferred and stored between Women's College Hospital, Markham-Stouffville Hospital and Cloud DX is currently being drafted and will be signed by all parties in the study prior to data transfer.

## Study Significance

The use of technology has the potential to provide clinical marker feedback to the patient and the clinical care provider (RM group), resulting in better disease control and better self-management skills and quality of life. Both programs have the potential to result in fewer face-to-face visits (outpatient and inpatient admissions), but the RM group may be more effective in that regard due to its ability to directly communicate with patients. The goal is to empower patients and improve their self-management skills leading to faster resolution of their health concerns resulting in fewer complications that may lead to outpatient and inpatient visits. This will increase institutional capacity to offer extended healthcare services to more patients.

## Risks

There is a risk that participants may feel uncomfortable with some items on some of the scales. If participants feel upset at any time during their participation in the study, they will be given the option to stop. In addition, there is a risk that some patients may feel more anxious about their disease once they know what their readings are or if their readings fall below threshold. We will advice patients to contact their health care provider or go to the emergency if they feel unwell or get anxious about their readings.

Participants who have extremely busy schedules and/or are trying to balance multiple personal, health and work demands may feel stress at the thought of taking time to participate in the study. At recruitment we will stress that participation is voluntary.

## Benefits

The findings of the study may contribute to a greater understanding of the effects of self and remote-monitoring in patients' ability to manage their disease. Patients will also receive the Cloud DX kit system for free and will keep it after completion of the study.

## Qualitative Realist Evaluation Protocol

### Background

In order to understand the contextual influences and practical processes of the Cloud DX platform implemented in MSH, we will use a qualitative process evaluation informed by realist methodology(Pawson, 2013). Realist Evaluation is a methodology used to “unpack the black box of implementation”(Marchal, van Belle, van Olmen, Hoerée, & Kegels, 2012), examining the actual actions required by those involved in implementation to ensure the Cloud DX platform is taken up and used by the clients and health care providers involved. This methodology enables a rigorous assessment of the contextual influences and strategies by which the application is adopted or rejected, enabling researchers to understand how and why the implementation succeeds or fails. Although we do not have resources to complete a full realist evaluation, our process evaluation incorporates principles of realist evaluation in order to provide insight into who benefits from the use of Cloud DX in what circumstances, and how. In order to best understand the mechanisms by which the technology was implemented, we will use a framework called the "Tool+Team+Routine" framework. This approach directs researchers' attention to the stakeholder who make up the team of people using the technology (including patients), the technology itself, and the new routines that need to be developed in order to successfully use the technology in everyday life. Interview questions will focus specifically on these elements in order to better illuminate the mechanisms involved.

### Objectives

The objective of the qualitative realist evaluation component of this study is to (a) understand how the Cloud DX platform is implemented in practice, (b) explore participants and members of the care team's perspectives of the usability and acceptability of the Cloud DX platform, and (c) examine the key issues associated with scaling of the Cloud DX platform in other settings across Ontario.

### Methods

The qualitative process evaluation will occur alongside the clinical trial described above, and will include semi-structured interviews with end users involved in the implementation process. Qualitative interviews will be conducted with participants and/or their caregivers, their healthcare providers, and hospital administrators/managers.  . Ten participants/caregivers, four healthcare providers, and three hospital administrators/managers will be recruited.

Qualitative interviews will include questions about (a) participants' experiences of learning about and using the technology; (b) changes to healthcare provider workflow required to effectively use the technology; (c) organizational changes required to support the technology; and (d) health system barriers and facilitators to effective implementation and evaluation (See Appendix 11A-C for Interview Guide). The interview guide includes specific questions related to the feasibility of the evaluations taking place, offering participants the opportunity to provide direct input into the implementation process. These qualitative interviews will be audio recorded and transcribed verbatim, and analyzed using thematic analysis strategies (Braun & Clarke, 2006; Pawson, 2013)  by the investigator team to identify key themes related to the implementation and evaluation of Cloud DX in actual contexts of health care delivery in Ontario.

### Recruitment

Patients

Potential participants will be identified prospectively by the clinical project specialist at MSH, based on whether they expressed interest during their consent process for the quantitative study. Individuals who express interest in the qualitative phase of the study will be directed to place a checkmark in the appropriate checkbox on the quantitative study consent form and their interest will be recorded in REDCap. The clinical project specialist at MSH will forward the names of the participants who are selected for the qualitative phase by the research coordinator at WCH, who in turn will contact the patients directly to follow-up, further explain the qualitative component of the study, provide the consent form, and schedule the interviews. Five patients from the RM and five from the SM group (and/or their caregiver if they were involved in taking measures) will be included in this stage. Healthcare providers involved in the care of participants and hospital administrators/managers will also be approached for participation in the qualitative portion of the study and interviewed upon study completion. These would include up to six healthcare providers involved in the care of patients who have participated in the quantitative part of the study and would include respirologists, RTs, and family doctors of the participants, and up to five hospital administrators/managers. These interviews will last no more than 60min each.

## Data Analysis

Qualitative interviews will be immediately transcribed into word documents and prepared for qualitative analysis. The findings of the qualitative data will be used to develop statements of the relationships between (a) key contextual factors, (b) the mechanisms by which they effect the implementation of the Cloud DX platform, and (c) the impact on the outcomes of the intervention themselves (in Realist Evaluation these statements are referred to as “Context-Mechanism-Outcome Configurations”)(Pawson, 2013).These statements will be used to revise a in order to accurately reflect the key contextual influences and practices that constitute the implementation process in actual health care settings in Ontario.

### Privacy and Confidentiality

All transcripts will be de-identified and data analysis will be aggregate in nature. Data stored electronically will be encrypted and password protected. Data will only be accessible to the investigators and hired research personnel who have received training in ethical and privacy principles.

Consent forms of study participants will be stored separately from the data files (transcripts and analytical worksheets). Only the Principal Investigator will have access to the consent forms, which will be kept in a locked cabinet for a period of 10 years. Once the dialogue from the interview audiotape(s) has been transcribed, the audiotape(s) will be destroyed. Password protected electronic copies of the transcribed audiotapes will be disseminated via email to the Investigative Team with the understanding that they will be destroyed after analysis. The hard copies of data (transcripts and analytical worksheets/notes) and informed consent forms will be shredded 10 years after study completion.

### Risks

There are no known harms associated with participation in this qualitative portion of the study.

There is a risk that participants may feel uncomfortable with items of discussion, or with being tape recorded. However, participants may withdraw from the study at any time or refuse to participate in any conversation that they find uncomfortable or embarrassing. If participants feel

upset at any time during their participation in the study, they will be given the option to stop. Participants will be given the option to have their de-identified data removed if they wish to.

Participants who have extremely busy schedules and/or are trying to balance multiple personal, health and work demands may feel stress at the thought of taking time to participate in an interview.

Benefits

The findings of the study may contribute to a greater understanding of the effects of self and remote-monitoring in patients' ability to manage their disease.

Conflicts of Interest
No known conflicts of interest.

## References


Braun, V., & Clarke, V. (2006). Using thematic analysis in psychology. *Qualitative Research in Psychology*, *3*(2), 77–101. https://doi.org/10.1191/1478088706qp063oa

Brown Connolly, N. E. (2014). A Better Way to Evaluate Remote Monitoring Programs in Chronic Disease Care: Receiver Operating Characteristic Analysis. *Telemedicine and E-Health*, *20*(12), 1143–1149. https://doi.org/10.1089/tmj.2014.0007

Choi, J. Y., Chung, H.-I. C., & Han, G. (2014). Patient outcomes according to COPD action plan adherence. *Journal of Clinical Nursing*, *23*(5–6), 883–891. https://doi.org/10.1111/jocn.12293

Clarke, M., Fursse, J., Connolly, N., Sharma, U., & Jones, R. (2017). Evaluation of the National Health Service (NHS) Direct Pilot Telehealth Program: Cost-Effectiveness Analysis. *Telemedicine and E-Health*. https://doi.org/10.1089/tmj.2016.0280

Cruz, J., Brooks, D., & Marques, A. (2014). Home telemonitoring in COPD: A systematic review of methodologies and patients' adherence. *International Journal of Medical Informatics*, *83*(4), 249–263. https://doi.org/10.1016/j.ijmedinf.2014.01.008

De San Miguel, K., Smith, J., & Lewin, G. (2013). Telehealth Remote Monitoring for Community-Dwelling Older Adults with Chronic Obstructive Pulmonary Disease. *Telemedicine and E-Health*, *19*(9), 652–657. https://doi.org/10.1089/tmj.2012.0244

Dewar, M., & Whit R. Curry, J. (2006). Chronic Obstructive Pulmonary Disease: Diagnostic Considerations. *American Family Physician*, *73*(4), 669–676.

Gershon, A. S., Guan, J., Victor, J. C., Goldstein, R., & To, T. (2013). Quantifying Health Services Use for Chronic Obstructive Pulmonary Disease. *American Journal of Respiratory and Critical Care Medicine*, *187*(6), 596–601. https://doi.org/10.1164/rccm.201211-2044OC

Government of Canada, S. C. (2014, March 19). Estimating the prevalence of COPD in Canada: Reported diagnosis versus measured airflow obstruction. Retrieved October 24, 2017, from https://www.statcan.gc.ca/pub/82-003-x/2014003/article/11908-eng.htm

Government of Canada, S. C. (2017, August 29). CANSIM - 105-0501 - Health indicator profile, annual estimates, by age group and sex, Canada, provinces, territories, health regions (2013 boundaries) and peer groups. Retrieved October 24, 2017, from http://www5.statcan.gc.ca/cansim/a26?lang=eng&id=1050501

Haesum, L. K. E., Soerensen, N., Dinesen, B., Nielsen, C., Grann, O., Hejlesen, O., … Ehlers, L. (2012). Cost-Utility Analysis of a Telerehabilitation Program: A Case Study of COPD Patients. *Telemedicine and E-Health*, *18*(9), 688–692. https://doi.org/10.1089/tmj.2011.0250

Jones, P. W., Harding, G., Berry, P., Wiklund, I., Chen, W.-H., & Kline Leidy, N. (2009). Development and first validation of the COPD Assessment Test. *The European Respiratory Journal*, *34*(3), 648–654. https://doi.org/10.1183/09031936.00102509

Jones, P. W., Quirk, F. H., & Baveystock, C. M. (1991). The St George's Respiratory Questionnaire. *Respiratory Medicine*, *85*, 25–31. https://doi.org/10.1016/S0954-6111(06)80166-6

Ko, F. W. S., Ngai, J. C. N., Ng, S. S. S., Chan, K., Cheung, R., Leung, M., … Hui, D. S. C. (2014). COPD care programme can reduce readmissions and in-patient bed days. *Respiratory Medicine*, *108*(12), 1771–1778. https://doi.org/10.1016/j.rmed.2014.09.019

Marchal, B., van Belle, S., van Olmen, J., Hoerée, T., & Kegels, G. (2012). Is realist evaluation keeping its promise? A review of published empirical studies in the field of health systems research. *Evaluation*, *18*(2), 192–212. https://doi.org/10.1177/1356389012442444

McBain, H., Shipley, M., & Newman, S. (2015). The impact of self-monitoring in chronic illness on healthcare utilisation: a systematic review of reviews. *BMC Health Services Research*, *15*(1). https://doi.org/10.1186/s12913-015-1221-5

McLean, S., Nurmatov, U., Liu, J. L., Pagliari, C., Car, J., & Sheikh, A. (2012). Telehealthcare for chronic obstructive pulmonary disease: Cochrane Review and meta-analysis. *Br J Gen Pract*, *62*(604), e739–e749. https://doi.org/10.3399/bjgp12X658269

Mittmann, N., Kuramoto, L., Seung, S. J., Haddon, J. M., Bradley-Kennedy, C., & FitzGerald, J. M. (2008). The cost of moderate and severe COPD exacerbations to the Canadian healthcare system. *Respiratory Medicine*, *102*(3), 413–421. https://doi.org/10.1016/j.rmed.2007.10.010

Pawson, R. (2013). *The science of evaluation: a realist manifesto*. London ; Thousand Oaks, Calif: SAGE.

Pedone, C., Chiurco, D., Scarlata, S., & Incalzi, R. A. (2013). Efficacy of multiparametric telemonitoring on respiratory outcomes in elderly people with COPD: a randomized controlled trial. *BMC Health Services Research*, *13*(1), 82.

Petkov, J., Harvey, P., & Battersby, M. (2010). The internal consistency and construct validity of the partners in health scale: validation of a patient rated chronic condition self-management measure. *Quality of Life Research*, *19*(7), 1079–1085. https://doi.org/10.1007/s11136-010-9661-1

Rixon, L., Hirani, S. P., Cartwright, M., Beynon, M., Doll, H., Steventon, A., … Newman, S. P. (2015). A RCT of telehealth for COPD patient's quality of life: the whole system demonstrator evaluation: COPD patient's quality of life. *The Clinical Respiratory Journal*, *11*(4), 459–469. https://doi.org/10.1111/crj.12359

Rojahn, K., Laplante, S., Sloand, J., Main, C., Ibrahim, A., Wild, J., … Johnson, K. I. (2016). Remote Monitoring of Chronic Diseases: A Landscape Assessment of Policies in Four European Countries. *PLOS ONE*, *11*(5), e0155738. https://doi.org/10.1371/journal.pone.0155738

Stenton, C. (2008). The MRC breathlessness scale. *Occupational Medicine (Oxford, England)*, *58*(3), 226–227. https://doi.org/10.1093/occmed/kqm162

Steventon, A., Bardsley, M., Billings, J., Dixon, J., Doll, H., Hirani, S., … Newman, S. (2012). Effect of telehealth on use of secondary care and mortality: findings from the Whole System Demonstrator cluster randomised trial. *BMJ*, *344*, e3874. https://doi.org/10.1136/bmj.e3874

Walters, J., Cameron-Tucker, H., Wills, K., Schüz, N., Scott, J., Robinson, A., … Walters, E. H. (2013). Effects of telephone health mentoring in community-recruited chronic obstructive pulmonary disease on self-management capacity, quality of life and psychological morbidity: a randomised controlled trial. *BMJ Open*, *3*(9), e003097. https://doi.org/10.1136/bmjopen-2013-003097

White, R., Walker, P., Roberts, S., Kalisky, S., & White, P. (2006). Bristol COPD Knowledge Questionnaire (BCKQ): testing what we teach patients about COPD. *Chronic Respiratory Disease*, *3*(3), 123–131. https://doi.org/10.1191/1479972306cd117oa

Zhang, Q., Liao, J., Liao, X., Wu, X., Wan, M., Wang, C., & Ma, Q. (2014). Disease knowledge level is a noteworthy risk factor of anxiety and depression in patients with chronic obstructive pulmonary disease: a cross-sectional study. *BMC Pulmonary Medicine*, *14*(1), 92. https://doi.org/10.1186/1471-2466-14-92

Zwerink, M., Brusse-Keizer, M., van der Valk, P. D. L. P. M., Zielhuis, G. A., Monninkhof, E. M., van der Palen, J., … Effing, T. (2014). Self management for patients with chronic obstructive pulmonary disease. *The Cochrane Database of Systematic Reviews*, (3), CD002990. https://doi.org/10.1002/14651858.CD002990.pub3
